# “Debriefing and Organizational Lessons Learned” (DOLL): A Qualitative Study to Develop a Classification Framework for Reporting Clinical Debriefing Results

**DOI:** 10.3389/fmed.2022.882326

**Published:** 2022-06-24

**Authors:** Méryl Paquay, Nadège Dubois, Anh Nguyet Diep, Gwennaëlle Graas, Tamara Sassel, Justine Piazza, Jean-Christophe Servotte, Alexandre Ghuysen

**Affiliations:** ^1^Department of Emergency, Quartier Hôpital, University Hospital of Liege, Liège, Belgium; ^2^Center for Medical Simulation of Liege, Quartier Hôpital, University of Liege, Liège, Belgium; ^3^Biostatistics Unit, Quartier Hôpital, University of Liège, Liège, Belgium; ^4^Namur Simulation, Namur-Liege-Luxembourg University College, Namur, Belgium

**Keywords:** clinical debriefing, teamwork, quality of care, patient safety, hospital management, debriefing, learning organization

## Abstract

**Background:**

The COVID-19 crisis has radically affected our healthcare institutions. Debriefings in clinical settings provide a time for the clinicians to reflect on the successes (pluses) and difficulties (deltas) encountered. Debriefings tend to be well-received if included in the broader management of the unit. The goal of this study was to develop a framework to categorize these debriefings and to assess its worthiness.

**Methods:**

A qualitative approach based on a grounded theory research method was adopted resulting in the “Debriefing and Organizational Lessons Learned” (DOLL) framework. Debriefings were conducted within two Emergency Departments of a Belgian University Hospital during an 8-week period. In the first step, three researchers used debriefing transcripts to inductively develop a tentative framework. During the second step, these three researchers conducted independent categorizations of the debriefings using the developed framework. In step 3, the team analyzed the data to understand the utility of the framework. Chi-square was conducted to examine the associations between the item types (pluses and deltas) and the framework's dimensions.

**Results:**

The DOLL is composed of seven dimensions and 13 subdimensions. Applied to 163 debriefings, the model identified 339 items, including 97 pluses and 242 deltas. Results revealed that there was an association between the frequency of pluses and deltas and the dimensions (*p* < 0.001). The deltas were mainly related to the work environment (equipment and maintenance) (*p* < 0.001) while the pluses identified tended to be related to the organization of the unit (communication and roles) (*p* < 0.001). With leadership's support and subsequent actions, clinicians were more enthusiastic about participating and the researchers anecdotally detected a switch toward a more positive organizational learning approach.

**Conclusion:**

The framework increases the potential value of clinical debriefings because it organizes results into actionable areas. Indeed, leadership found the DOLL to be a useful management tool. Further research is needed to investigate how DOLL may work in non-crisis circumstances and further apply the DOLL into incident reporting and risk management process of the unit.

## Introduction

The COVID-19 crisis has affected our healthcare institutions (1). Hospitals had to face major economic (2), structural (3) and organizational (4) challenges in a high workload, uncertain and constantly changing environment. This raised concern about impacts on quality and patient safety (5) and a notable psychological burden on professionals (6). Initiatives, such as the Circle-Up process, were developed to help deal with the consequences of the crisis (7). Circle-up is a process aiming to connect team members and to facilitate organizational learning based on three activities: before shift briefings, during shift peer check-ins and post shift debriefings. The before shift briefing is to decide on a work plan, clarify procedures, roles and responsibilities and to create a team spirit. During a shift, peer check-ins help report on the situation, update team situation awareness and offer support to the clinicians. Post shift debriefings provide a time for the participants to reflect on shift successes and difficulties (7). During the post-shift debriefing, the participants gather to review the entire shift. The value of these team debriefings in the clinical environment appears to be twofold. First, they help share information within the clinical team and with leadership to solidify operations where things were working well and to improve things not working as well as they could (7–12). Secondly, clinical debriefings might help to provide a safe place for controlled psychological support, situational awareness, workload management and operational support (8).

Past research resulted in the development of guidance to conduct clinical debriefings (8, 9, 13, 14). During COVID-19, the *Debriefing In Situ COVID-19 to Encourage Reflection and Plus-Delta in Healthcare After Shifts End* (DISCOVER-PHASE) was specifically developed to implement a standardized clinical team debriefing program (15). The DISCOVER-PHASE seeks to analyze all types of events encountered and was designed to be used at the end of shift (13). It employs a plus/delta method. Indeed, the importance of learning not only from failure but also from success and leadership's wholehearted and visible commitment to act on things that are going well (pluses) and things that need improvement (deltas) appears essential.

Clinical debriefings are a relatively new area of practice. Studies have investigated the most effective method for conducting debriefings in the clinical environment (8, 9, 15–18). However, evidence regarding the frequency of post-shift debriefings and their embedding in a global strategy is scarce. Based on the scarce findings to date, debriefings appear to hold promise to be a keystone for promoting a learning organization culture and for triggering quality and safety improvement (9, 19). In that perspective, providing feedback on the teams' input is an essential programmatic element that could garner support from the clinician teams and for organizational learning. Strengths and opportunities for improvement should be addressed and comparisons with experiences made to identify what remedial actions should be undertaken and the underlying thought processes for those actions. Such commitments could prove difficult to achieve in the absence of structured and standardized tools for the appropriate processing of the data collected during these debriefings. To date, no formal classification system has been developed to organize debriefing data and allow the results to be easily integrated into a unit's internal and external management, resourcing, and coordination of the department. Therefore, we designed the present study with the aim to develop a framework to categorize results from regularly implemented post-shift debriefings into a useful and actionable framework (DOLL) and to perform an initial assessment of its feasibility and utility in the real world of clinical care. With this idea in mind, the framework was employed during a two-month period of the COVID-19 pandemic.

## Materials and Methods

### Study Design

We used a three-step approach to conduct this study. The first step relied on an adapted qualitative approach based on grounded theory research to construct the DOLL framework (20). It used content analysis of post-shift debriefings in the clinical environment to move beyond description and to generate a theory grounded through meaningful categories of information. The second step was comprised of implementing post-shift debriefings and categorizing the debriefing content. The third step was to analyze the quality of the data and reflect on the utility of the framework. Standards for Reporting Qualitative Research (SRQR) (21) were used to help guide the study.

### Study Site

Data was collected from two Emergency Departments (ED) of a single Belgian University teaching hospital with two geographically separated facilities: Main and Satellite. The Main facility is a tertiary care hospital located in a suburban area, while the Satellite is an urban secondary hospital. The ED from the Main facility was raised under the cultural umbrella of a Public University Teaching Hospital while the second ED history started as part of a private clinic that was merged with the Main Hospital. The two sites combine an annual ED census of approximately 100,000 patients: with the Main handling approximately 57% and the Satellite handling 43%. The department employs approximately 50 physicians and 120 nurses. Nurses are assigned to Main or Satellite while physicians are scheduled at both sites.

### Data Collection

Data was collected on 163 debriefings conducted during 8 weeks, from March 10 to May 10 of 2020. The debriefings were led in French. The median number of attendees per debriefing was 5 (IQR: 4–6) and remained stable over the observed period. These debriefings were performed after morning and afternoon shifts among medical and nursing members of the ED in the two facilities. Clinical debriefings had a median duration of 10 min. The durations were longer in the first 2 weeks and decreased by over 50% in the latter half of the study period. The same facilitator led most debriefings (142 of the 163) while five other facilitators performed the remaining debriefings (21 of 163). All debriefers had a clinical background and previous training in Debriefing With Good Judgment (22) and the Plus-Delta method. Of the 163 debriefings, 94 were held at the Satellite and 93 were held in the Main facility. DISCOVER-Phase tool was used to collect the following post-shift debriefing data: date, site, shift, members, “plus/delta” and initiatives proposed. Before the project was undertaken, the facilitator (MP) spent one immersive month becoming familiarized with the then-current ED structures, procedures, equipment, and teamwork habits.

### Data Analysis

#### Building the Classification Framework

**Figure 1:**
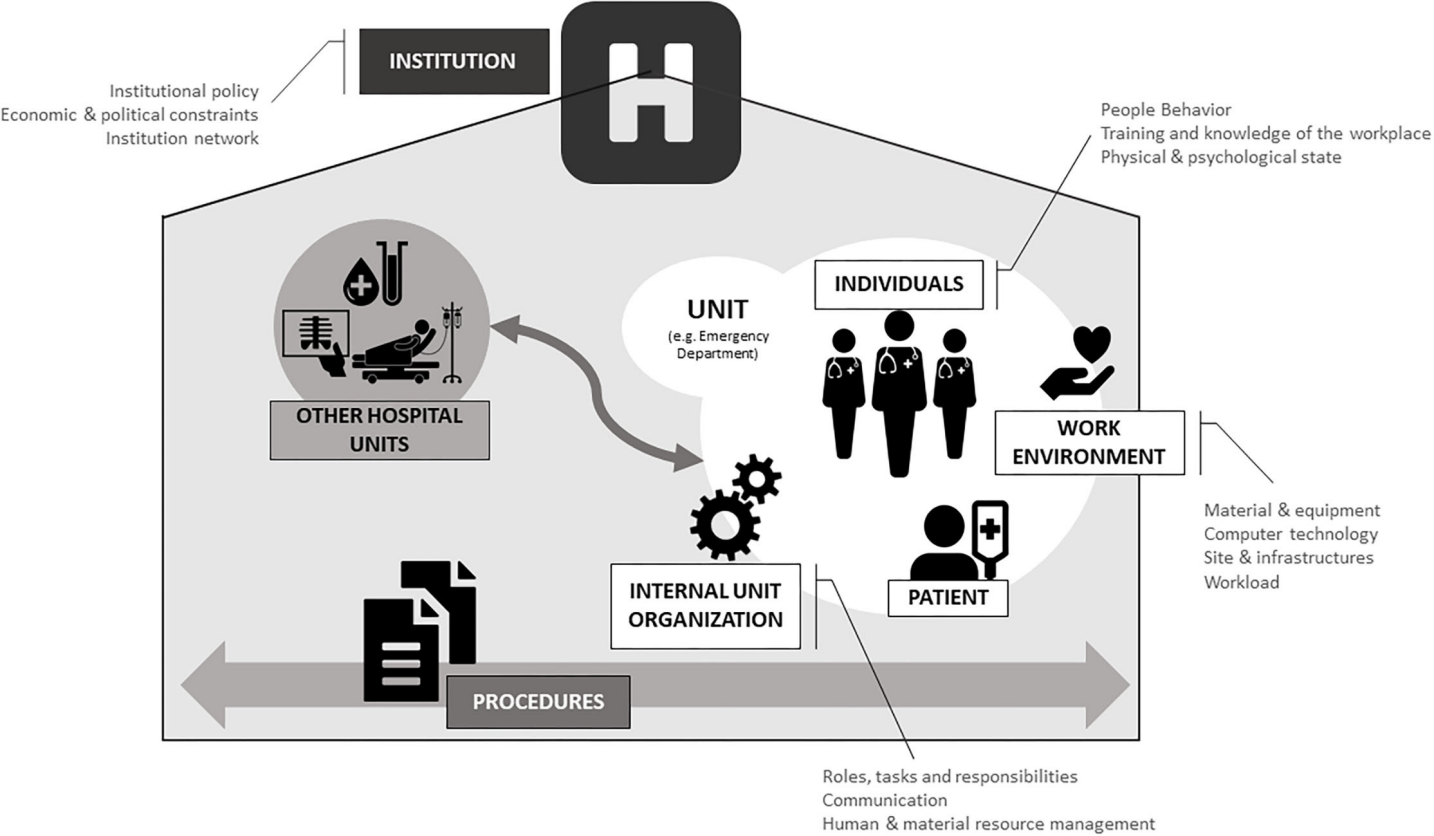
Paquay-Ghuysen Debriefing and Organizational Lessons Learned (DOLL) © framework.

Three researchers (MP, GG, TS) from different occupational categories (quality and safety, process manager, and psychologist) read all the transcribed debriefing reports. The different backgrounds provided a multidimensional view for interpreting results. The researchers further self-reflected about a classification framework based on their specific background and the transcribed debriefings. This step led to the identification of three candidate frameworks: (a) “Framework for analyzing risk and safety in clinical medicine” (23), (b) Ishikawa/Fishbone diagram (24) and (c) the “European framework for psychosocial risk management” (25). Once the frameworks were chosen, each researcher refined the existing categories for their candidate framework with the topics raised in all 163 debriefings. Then, the researchers shared and compared their frameworks together to decide which categories were relevant and should be retained or discarded. This broad-then-narrowing of the categories was followed by a comprehensive in-person discussion that resulted in the “Debriefing and Organizational Lessons Learned (DOLL)” framework (Figure 1).

#### Debriefings Categorization and Content Analysis

The researchers started the analysis by individually reading the transcripts several times to become familiar with the data. The research team started collectively the sorting on a subset of the data to reach a reasonable understanding and consistency of the process. Then, the researchers worked independently to classify each item as “plus” or “delta” and then placed items into the DOLL framework. Resulting individual analyses were then compared and discussed by the research team until consensus was reached. For that purpose, the three researchers exposed each item and its classification. When a classification was not the same between the researchers, they reanalyzed the item and collectively reached 100% reconciliation through discussion, curiosity, frames exploration and clarification using a process similar to a learning pathway grid (26). Due to their different professional backgrounds, discussion helped sharing individual perspectives and understandings.

### Statistics

Descriptive statistics were performed to summarize the frequencies and percentages of the pluses and deltas in each dimension of the DOLL. Chi-square was conducted to examine the association between the type of items (plus or deltas) and the Dimensions followed by *post-hoc* tests using adjusted standardized residuals. Fisher exact test was employed. Furthermore, z-test for independent proportions was applied to investigate if there were any significant differences in the number of plusses and deltas across the different dimensions. Bonferroni correction was used for all *post-hoc* comparisons.

### Ethical Considerations

This study was approved by the ethical committee of the Liege University Medicine Faculty with the reference number 2020/252. All participants gave informed consent to participate in the study.

## Results

### Classification Framework Development

The classification framework developed is depicted in Figure 1. It is composed of 7 Dimensions and 13 Subdimensions. The agreed upon Dimensions and Subdimensions are described in Table 1.

**Table 1:** Debriefing and Organizational Lessons Learned (DOLL) framework dimensions and subdimensions.

**Dimensions**	**Subdimensions**	**Description**
Patient		Refers to the patient's clinical condition, social and familial environment.
Individuals	People behavior	Relates to personality and belonging to a specific group of professionals
	Training and knowledge of the workplace	Refers to competencies, procedures training program and understanding of the immediate environment
	Physical and psychological state	Deals with mental or physical condition like tiredness or stress.
Internal unit organization	Roles, tasks and responsibilities	This subdimension defined by the objectives set by the team, task planning and roles distribution.
	Communication	Refers to oral and written communication, communication tools and reporting of information
	Humans and materials resources management	Relates to how resources are used and managed within the unit
Procedures		Refers to the adequacy, efficiency or clarity of the unit and hospital's clinical and organizational procedures
Work environment	Material and equipment	Considers how the unit is supplied by the hospital
	Computer technology	Refers to the availability and functioning of the computer system, user interface, guidelines and system maintenance
	Site and infrastructure	Relates to physical structure of the unit including architecture, engineering, the structural issues and availability of space
	Workload	Is defined by the type and quality of unit workload.
Other hospital units		Refers to the impact other units or departments decisions on the unit of interest
Institution	Institutional policy	Refers to the hospital's organization, governance and policies
	Economic and political constraints	Is defined by regulatory and financial aspects impacting the unit
	Institutional network	Considers the link to other organizations, programs and levels of government

### Debriefing Categorization Using DOLL

The 163 debriefings were evenly distributed between the two sites. Out of these debriefings, 339 items were identified including 97 pluses (29%) and 242 deltas (71%) (Table 2). During the “compare and discuss” process, classification was not the same for 64 items (18%) and needed a second collective analysis to reach 100% consensus.

**Table 2:** Percentages of Plus and Delta based on the “Debriefing and Organizational Lessons Learned (DOLL)” framework dimensions.

**Dimensions**	**Subdimensions**	**Percentage of Plus**		**Total Plus** **n (%)**	**Percentage of Delta**		**Total Delta** **n (%)**	**Total n (%)**
		**Main n (%)**	**Satellite n (%)**		**Main n (%)**	**Satellite n (%)**		
Patient	**Total**	**0 (0)**	**0 (0)**	**0 (0)**	**2 (1)**	**0 (0)**	**2 (1)**	**2 (1)**
Individuals	**Total**	**20 (6)**	**17 (5)**	**37 (11)**	**17 (5)**	**27 (8)**	**44 (13)**	**81 (24)**
	*People behavior*	*14 (4)*	*12 (4)*	*26 (8)*	*3 (1)*	*3 (1)*	*6 (2)*	*32 (10)*
	*Training and knowledge of the workplace*	*6 (2)*	*4 (1)*	*10 (3)*	*11 (3)*	*21 (6)*	*32 (9)*	*42 (12)*
	*Physical and psychological state*	*0 (0)*	*1 (0)*	*1 (0)*	*3 (1)*	*3 (1)*	*6 (2)*	*7 (2)*
Internal unit organization (Emergency Department)	**Total**	**20 (6)**	**22 (6)**	**42 (12)**	**15 (5)**	**15 (4)**	**30 (9)**	**72 (21)**
	*Roles, tasks and responsibilities*	*10 (3)*	*15 (4)*	*25 (7)*	*2 (1)*	*3 (1)*	*5 (2)*	*30 (9)*
	*Communication*	*5 (2)*	*4 (1)*	*9 (3)*	*1 (0)*	*3 (1)*	*4 (1)*	*13 (4)*
	*Humans and materials resources management*	*5 (1)*	*3 (1)*	*8 (2)*	*12 (4)*	*9 (2)*	*21 (6)*	*29 (8)*
Procedures	**Total**	**1 (0)**	**9 (3)**	**10 (3)**	**23 (7)**	**26 (7)**	**49 (14)**	**59 (17)**
Work environment	**Total**	**6 (2)**	**1 (0)**	**7 (2)**	**39 (11)**	**47 (14)**	**86 (25)**	**93 (27)**
	*Material and equipment*	*2 (1)*	*0 (0)*	*2 (1)*	*18 (5)*	*13 (4)*	*31 (9)*	*33 (10)*
	*Computer technology*	*1 (0)*	*0 (0)*	*1 (0)*	*14 (4)*	*18 (5)*	*32 (9)*	*33 (10)*
	*Site and infrastructures*	*1 (0)*	*1 (0)*	*2 (0)*	*6 (2)*	*14 (4)*	*20 (6)*	*22 (6)*
	*Workload*	*1%*	*0%*	*1%*	*0%*	*1%*	*1%*	*2%*
Other hospital units	**Total**	**1 (0)**	**0 (0)**	**1 (0)**	**8 (2)**	**5 (2)**	**13 (4)**	**14 (4)**
Institution	**Total**	**0 (0)**	**0 (0)**	**0 (0)**	**11 (3)**	**7 (3)**	**18 (6)**	**18 (6)**
	*Institutional policy*	*0 (0)*	*0 (0)*	*0 (0)*	*10 (3)*	*3 (1)*	*13 (4)*	*13 (4)*
	*Economic and political constraints*	*0 (0)*	*0 (0)*	*0 (0)*	*0 (0)*	*2 (1)*	*2 (1)*	*2 (1)*
	*Institution network*	*0 (0)*	*0 (0)*	*0 (0)*	*1 (0)*	*2 (1)*	*3 (1)*	*3 (1)*
All dimensions	**Total**	48 (14)	49 (15)	**97 (29)**	115 (34)	127 (37)	**242 (71)**	**339 (100)**

*Total Pluses and Deltas = 339*.

*Main n = 163 /Satellite n = 176*.

Pluses were equally distributed between the two sites (49% Main−51% Satellite). Due to the presence of cells with zeros, Fisher exact test was performed. The result revealed that there was an association between the frequency of pluses and deltas and the different Dimensions (*p* < 0.001). *Post-hoc* examination using adjusted standardized residuals and Bonferroni correction (i.e., value < 0. 0036 being significant) revealed that there were more pluses expected in the Dimension Individuals (*p* < 0.001) and Internal unit organization (*p* < 0.001). On the contrary, there were more deltas than expected in the Dimension Work environment (*p* < 0.001).

Z-test for independent proportions using Bonferroni correction indicated that there were significantly less deltas in the Dimension Individuals (54.3%, *p* < 0.05) compared to that of Other hospital units (93%), Work environment (93%), Institution (100%), and Patient (100%). There were significantly less deltas in the Dimension Internal unit organization (42%, *p* < 0.05) compared to that of Procedures (83%), Work environment (92%), Other hospital units (93%) and Institution (100%).

Within the pluses, there were significantly more pluses in the Dimensions Individuals (46%, *p* < 0.05) and Internal unit organization (58%, *p* < 0.05) than that of Other hospital units (7%), Work environment (8%), and Procedure (16%). Results are presented in Table 3.

**Table 3:** Number of deltas and pluses across different dimensions.

**Dimension**	**Delta** **n (%)**	**Plus** **n (%)**	**Total**
Patient	2 (100)	0 (0)	2
Individuals^a^	44 (54)	37 (46)	81
Internal unit organization^a^ (Emergency Department)	30 (42)	42 (58)	72
Procedures	49 (83)	10 (17)	59
Work environment^b^	86 (92)	7 (8)	93
Other hospital units	13 (93)	1 (7)	14
Institution	18 (100)	0 (0)	18
**All dimensions**	**242**	**97**	**339**

*Post-hoc chi-square indicate*.

^a^*More pluses in the Dimensions Individuals (p < 0.001) and Internal unit organization (p < 0.001)*.

^b^*More deltas in the Dimension Work environment (p < 0.001)*.

### Debriefing Content Analysis Using DOLL

Table 4 presents the Dimensions, Subdimensions and examples of what participants stated for each Dimension in the DOLL.

**Table 4:** Debriefing and Organizational Lessons Learned (DOLL) framework dimensions and subdimensions examples.

**Dimensions**	**Description**	**Plus and Delta Examples**
Patient	Refers to the patient's clinical condition, social and familial environment.	**Delta**: *≪ A patient in the drive with red flag to be examined refused to get out of the vehicle and left ≫*
Individuals	**People behavior**: Relates to personality and belonging to a specific group of professionals	**Plus:** “*Colleagues can rely on each other. There is a good working atmosphere”*
	**Training and knowledge of the workplace**: Refers to competencies, procedures training program and understanding of the immediate environment	**Delta:** “*An improvement could be made regarding nurses from other units. They are not briefed and do not know the procedures. One of them was particularly anxious, as she was not at all familiar with the emergency room. We should give these nurses at least 24 hours' notice to give them the opportunity to read the procedures, encourage continuity as much as possible (the same people coming to help) and ensure that their skills profile is correct.”*
	**Physical and psychological state**: Deals with mental or physical condition like tiredness or stress.	**Delta:** “*Colleagues are “dropping like flies” around us, it is not a fear related to a risk of transmission to our children, but rather related to the loss of efficiency and skills if they are no longer used”*
Internal unit organization	**Roles, tasks and responsibilities:** This subdimension defined by the objectives set by the team, task planning and roles distribution.	**Plus:** “*A good routine has been established between the team members and we are getting used to our new missions. It's great to work in these conditions.”*
	**Communication:** Refers to oral and written communication, communication tools and reporting of information	**Plus:** “*Overall, the team notes a clear improvement in medical-nursing communication in the village [Covid structure] compared to the beginning thanks to greater exchange of information, feedback, closed loop communication”*
	**Humans and materials resources management:** Relates to how resources are used and managed within the unit	**Delta:** “*The work is lighter and there is a feeling of imbalance of forces. The team wonders if it would not be necessary to transfer a nurse to the traditional emergency department because they have the impression of being under-utilized.”*
Procedures	Refers to the adequacy, efficiency or clarity of the unit and hospital's clinical and organizational procedures	**Delta:** “*This weekend, COVID unit received several pregnant women from the maternity ward for smears. Sometimes they came with labels, and at other times only with the request. In addition to the fear of contaminating them and making them wait a long time, the nurses think that there should be a clear procedure to avoid administrative problems and even long waiting times”*
Work environment	**Material and equipment:** Considers how the unit is supplied by the hospital	**Delta:** “*The printers are all located in the consultation room. If a medical prescription is printed, it is automatically considered contaminated. Proposal: placing a printer in the corridor would avoid this problem*
	**Computer technology:** Refers to the availability and functioning of the computer system, user interface, guidelines and system maintenance	**Delta:** “*Recurring problems with the IT department. Called this day at least 3 times since the morning because it was impossible to print from the PCs. Finally, nobody ever came... This is a problem for transfusions because it is impossible to print the labels to be scanned. You have to call the colleagues from the [standard] ED to get these labels, which is very tiresome.”*
	**Site and infrastructure:** Relates to physical structure of the unit including architecture, engineering, the structural issues and availability of space	**Delta:** “*Problem with the turf. The net would sink. A carpenter came but can't do much. This causes difficulties when patients are in wheelchairs (difference in level)”*
	**Workload:** Is defined by the type and quality of unit workload.	**Delta**: ≪*Workload is heavier. There is an overall fatigue within the team after an 8-hour shift during which we had to concentrate considerably more. Everything takes longer ≫*
Other hospital units	Refers to the impact other units or departments decisions on the unit of interest	**Delta:** “*There were a lot of people waiting at the drive opening, requiring an early opening. It's like that every day. The drive is busy at the opening and during the morning and then much quieter the rest of the day. Most of the patients who come for a pre-hospitalization smear test say that they were told by the anesthesiologist or their doctor to come early in the morning, which contributes to the overloaded opening of the drive”*
Institution	**Institutional policy:** Refers to the hospital's organization, governance and policies	**Delta:** “*The hospital visits will be allowed again starting this Tuesday. The team is concerned about not having guidelines. They are wondering what the process is for visitors to accompany patients admitted to the ED. Are visitors allowed in the ED?”*
	**Economic and political constraints:** Is defined by regulatory and financial aspects impacting the unit	**Delta:** “*More on the financial side, teams noted a lack of equity between people on temporary lay-off, who would receive 90% of their salary and caregivers who, if they are sick, would receive less. In addition, statutory workers would lose legal paid vacation days.”*
	**Institutional network:** Considers the link to other organizations, programs and levels of government	**Delta:** “*Patients coming from nursing homes were, in most cases, patients at the end of life with clear notification in their file. Managing these patients is long and complicated (confusion) and the structure is not suitable for providing serene end-of-life care. At a time, 4 of the 5 patients in one area of the ED were nursing home patients at the end of life. The hospital needs to meet with the nursing home network to find out how to collaborate.”*

#### Patient

Two items were associated with this dimension and were exclusively deltas. Teams highlighted the difficulty of taking care of extreme and rare clinical cases or patient violence despite having all the procedures and competencies at their disposal.

#### Individuals

This dimension was mostly marked by the pluses in the Subdimension People behavior (70.3%) and by the deltas in the Subdimension Training and knowledge of the workplace (72.7%). Related to the Dimension, teams positively reported behaviors of solidarity, mutual aid, commitment to the work and the team. On the other hand, teams expressed difficulty in getting familiar with the environment and procedures, especially when it concerned nurses on assignment to the ED from other units. Lastly, Physical and psychological state was mostly characterized by deltas (85%) with participants expressing their stress.

#### Internal Unit Organization

This Dimension included greater pluses (58%) than deltas (41%). Pluses focused on the Roles, tasks, and responsibilities (60%) while Humans and materials resource management was mainly defined by deltas (70.0%). Teams complimented the established organization, particularly task and role assignment between physicians and nurses. However, the management of teams and materials was problematic for the staff. In this respect, participants explained that the workload was more unpredictable than usual and that it was difficult to anticipate how to adequately allocate human resources. On the other hand, communication was more often reported as positive (69%), which referred mainly to the quality of medical-nursing communication.

#### Procedures

This Dimension was comprised almost exclusively by deltas (83%). Teams highlighted the changes on an almost daily basis in procedures or the absence of procedures for frequently encountered problems.

#### Work Environment

This Dimension was also largely described by deltas (93%). Comments by participants revealed that Material and equipment (36%), Computer technology (37%) and Site and infrastructures (23%) were the three Dimensions that mostly needed to be improved.

#### Other Hospital Units

This Dimension consisted almost exclusively of deltas (92.8%). Teams pointed to a lack of coordination between the ED and other units and a lack of available consultation in the decision-making process. The latter had a negative impact on the ED. Laboratory, medical imaging and pre-anesthetic consultations were cited as the most problematic.

#### Institution

The Dimension Institution was only defined by deltas (100.0%). Teams mainly emphasized the lack of institutional policy (72.2%) related to important issues (e.g., visits, staff screening, patient flowchart) and communication strategy.

## Discussion

As clinical debriefing programs gained in popularity, recent research has been conducted to propose guidelines for debriefing implementation (14) and to engage leaders in the process (9). Recent works also helped to set boundaries in terms of debriefing objectives to ensure efficacy (8). Such results promoted debriefing as a learning tool (reflection on the work done and processes) instead of debriefing as a treating tool (mental health exploration). Using a qualitative approach, we aimed to develop a classification framework and to categorize results from regularly implemented post-shift team debriefings. The DOLL framework is composed of seven dimensions and 13 subdimensions. To the best of our knowledge, this is the first study developing a classification framework for debriefings in a clinical environment. During the first Belgian wave of the COVID-19 crisis, the application of this framework revealed interesting perspectives, allowing for a more structured and standardized analysis of debriefings. Such analysis identified actionable areas and insights, perfectly in line with the experience of the teams, and offered a useful management tool for the leadership.

Considering the debriefing categorization using the DOLL, we found that some Dimensions of the framework were less used than others. The “Patient” Dimension in this study only included two items, probably because very few items mentioned during the debriefings were exclusively patient-related. We also found that the “External unit organization” dimension could be merged into the “Institution” Dimension. This raises questions about the relevance of keeping these Dimensions in the framework. However, the DOLL framework was tested only once and during a short period of the COVID-19 crisis. Rather than eliminating these Dimensions at the time being, it would be worthwhile to further test the current DOLL framework in a non-Covid context to assess their relevance before any modifications.

The analysis of the debriefing content using the DOLL framework reveals differences in the number of plus and delta comments, with more deltas compared to pluses and, interestingly, several inequalities in the distribution of pluses and deltas among Dimensions.

Regarding plus and delta classification, our results highlight the predominantly negative impact of the *work environment* on the clinicians and their capacity to provide effective and efficient care. A shortage of personal protective equipment (PPE) and daily materials or drug supply challenged the clinicians. (27). According to our results, the fear of infecting family and friends was described as a major stress factor for many participants (28). These findings highlight the need to implement strategies to manage emergency reserves, and to determine situations in which PPE is required and when PPE use may interfere with performance. Along with material availability, the results emphasize the need for efficient supporting services such as IT support, mechanical and electric maintenance, accessibility, etc. Indeed, few studies have identified the latter themes in their findings or chosen these issues as a research topic but those who did have pointed out the importance of a proactive IT Department with a clear strategy (29) and the urgent need to improve interoperability and intuitive user interfaces (30). As part of the healthcare process, support services such as maintenance have the potential to markedly improve patient care when they are immediately available, sensitive to ongoing care priorities and complement an interdisciplinary environment.

*Clinician teamwork behaviors* were a frequent topic. Debriefings revealed that those behaviors were positively valued. In the face of objectively difficult circumstances, clinicians commented on having greater motivation, engagement and solidarity than before the crisis. Past research has also highlighted that despite the psychological burden, personal risk and work overload, nurses tend to have high work engagement (31) and uphold work values (32). Our data illustrate how the COVID-19 crisis, complemented with post shift debriefings, offered meaning, support and a common cause to clinicians. This raises a fundamental question about how, in the context healthcare's ever-increasing stressful, high-risk environment, we can find ways to support clinicians to avoid burnout and maintain commitment to a high level of patient care. The DOLL framework and its guidelines have been developed and successfully implemented in this experiment. Further testing and refinement of the DOLL is encouraged as it appears to warrant further work (33, 34).

Based on participant remarks, unit management and coordination appeared to improve throughout the two-month data collection. Participants noticed that the debriefing system and the feedback communication loop created with the ED strategic committee were highly effective. The committee was comprised of influential and leadership-designated nurses and physicians. Issues reported during debriefings were systematically brought to the attention of the committee, which set up and communicated action plans in coordination with ED clinicians. This closed loop communication coupled with positive actions was key to success. It provided a sense of empowerment; participants felt listened to and that their point of view was taken seriously. As also stated in previous studies, participants frequently verbalized their approval and willingness to continue participation of the debriefings because they could clearly notice changes that started in the debriefings (9). Similarly, previous work has reported participant satisfaction about debriefings regarding emotional support and organizational learning (7, 15, 35). As debriefing programs gained in popularity, research has been conducted to propose guidelines for implementation (14) and guidelines to engage leaders in the process (9). Recent guidelines also helped to put boundaries in terms of debriefing objectives to ensure efficacy (8). These results promoted debriefing as a learning tool (reflection on the work done and processes) instead of debriefing as a treating tool (mental health exploration). Study participants have also reported that they were more likely to attend debriefings when organization and work were discussed rather than too much emphasis on feelings and mental health. Besides participant satisfaction, what is relatively new is that department leadership was increasingly enthusiastic about the debriefing project as they came to realize how the debriefings and the DOLL were becoming a powerful and effective management tool. Such result points out that debriefings themselves are valuable insofar as a comprehensive structure is implemented to provide effective follow-ups. Perhaps as best practices are developed for clinical debriefing, guidelines for leadership need to be included, e.g., closing the loop on good things that happen as well as the things leaders are working to improve, having some resources to address problems, devoting resources to structuring results of clinical debriefings, etc. Furthermore, there are some of the lessons-learned here that may hold promise for processes such as incident reporting or patients' satisfaction.

In terms of *procedures and associated processes*, the COVID-19 pandemic required committed effort to adapt procedures and practices under the paradox of quick and safe (36). We found that participants from the present study often reported a lack of organizational procedures usually because the situation was so novel and dynamic in its nature. This issue has already been raised by previous research such as screening at the ED entrances (37), rapid PCR testing for COVID-19 patients (38, 39) or patient handovers (40). Regarding routine, well-established clinical and technical procedures, which were mostly available, teams reported a lack of dissemination and training notwithstanding, especially with regard to the unfolding COVID-19 crisis. Indeed, the availability of established procedures or protocols does not guarantee proper use (41). Poor mastery of procedures logically leads to greater clinician and patient risk such as increased exposure and contamination (42) and reduced effectiveness. Different strategies to enhance effective implementation of procedures were also raised as pluses by the participants. For example, they became interested in having briefings at the beginning of the shift to go through new procedures. Pre-shift briefings seem to have helped master clinical and teamwork skills (such as anticipating and planning, task responsibility, role allocation, etc.), likely allowing for enhanced team and individual performance while maintaining a good margin for patient safety as previously reported (43). Simulation-based training was also pointed out as a major asset by the participants. Participants described simulation as one of the best methods to master clinical and teamwork skills in a safe environment (44). Indeed, the use of simulation has been highly promoted during the COVID-19 crisis to practice, implement, improve procedures (45), to validate procedures (46) and to improve crisis resource management (47, 48).

Regarding the framework itself, the lack of a common taxonomy and the inconsistencies in the results reported in past studies on debriefings impairs a reliable focus on comparisons and trends in the scientific literature (49). The adoption of a common reporting framework for clinical debriefings has the potential to provide a potentially powerful management tool and allow meaningful comparisons even if departments use different debriefing tools or programs. In our experience, regarding the classification of the debriefings, the framework developed allowed for comparisons between Satellite and Main, or Dimensions and the Subdimensions, which helped to refine and focus the larger concepts presented by the Dimensions. Moreover, unlike most classifications used for safety and quality improvement, the framework was developed with the desire of reporting not only issues but also successes. For years, work to improve safety was mainly based on the analysis of undesirable or unsafe written incidents and issues (Safety I) (50). However, that strategy sometimes led to poor outcomes (16). Safer care needs a better and deeper understanding of daily processes, including building on successes (Safety II) and in such context, debriefings can help to identify, analyze, and reinforce the positive practice (9).

Besides the framework itself, its practical use has outlined major benefits and some negative points. In our opinion, the usefulness of the framework further lays in its capacity to clearly illustrate the essence and value of debriefings. This has been a great support for leaders as it helped them to allocate resources and priorities more efficiently. In addition, analyzing the debriefings through main categories helped the leaders to think in terms of processes and workflow rather than problem-solution. The ED found itself developing an ongoing quality improvement programs vis-à-vis the clinical debriefings coupled with favorable action. Before the framework, each plus or delta was reported and addressed individually. When using the framework, more links were made between debriefings and their evolving content over time. Using the framework has also made reporting and traceability more affordable. For future use, resource people could be identified for each Dimension to address the issues raised and to work further on improvement, just as the practice for incident reports or risk assessment. However, to fully benefit from the framework, a specific and visible reporting and management platform would be needed.

## Limitations

The debriefing program was part of the COVID-19 quality improvement strategy in our ED. Two different hospitals comprised the experimental sites, but they are located in one larger geographical locale. As the DOLL classification framework and its implementation was based on these data, further research is needed to test the model in different localities and contexts. Interpretation bias is also common in qualitative studies during data collection and analysis. Since most debriefings were conducted by the same trained investigator, content may have been unintentionally affected to some extent. To limit interpretation bias, classification was independently achieved by three different investigators from three different backgrounds and then reconciled to 100% agreement. If such programs are to be implemented, more trained debriefers should be involved. To date, scientific evidence is scarce as to how a debriefer's personality, background or debriefing technique may influence the outcomes of clinical debriefings. Lastly, because leadership responses to debriefings and the DOLL were not systematically quantitatively or qualitatively captured, this is another area that could be further investigated.

## Conclusions

We developed a classification framework to help analyze and utilize the content of post-shift clinical debriefings. The practical aim of the project was to maintain a commitment to quality improvement, ensure effective patient care and provide clinicians with ongoing support from colleagues and leadership. Testing the DOLL framework in two different EDs revealed how identifying positive and negative aspects of performance within specific dimensional frames and combining them with guidelines for using the categorized results was a useful and well-received technique. Using such framework allows to increase the potential value of debriefings in the clinical environment by integrating the information gathered into a broader strategy. Debriefings coupled to the framework offer a useful management and leadership tool within a specific department. Links with other existing processes, such as risk management, incident management or patients' complaints, could be investigated to fully take advantage of the potential of debriefings coupled with the DOLL. Our experience convinced us that routinely implementing the technique as part of our department's standard operating procedures has notably enhanced our ongoing practices and commitment to our patients and clinicians.

## Data Availability Statement

The original contributions presented in the study are included in the article/supplementary material, further inquiries can be directed to the corresponding author.

## Author Contributions

MP conceived and designed the study, realized data analysis, and wrote the first draft. ND facilitated data transcription and realized critical revision of article for important intellectual content. GG helped with study design, facilitated data transcription, and performed data analysis and interpretation. AD performed data analysis and made critical revision. JP and J-CS realized data collection and editing. AG conceived and designed the study, critical editing, and performed supervision. All authors contributed to the manuscript revision, read, and approved the submitted version.

## Conflict of Interest

The authors declare that the research was conducted in the absence of any commercial or financial relationships that could be construed as a potential conflict of interest.

## Publisher's Note

All claims expressed in this article are solely those of the authors and do not necessarily represent those of their affiliated organizations, or those of the publisher, the editors and the reviewers. Any product that may be evaluated in this article, or claim that may be made by its manufacturer, is not guaranteed or endorsed by the publisher.
